# Early Life Maltreatment and Adolescent Interpretations of Ambiguous Social Situations: Investigating Interpersonal Cognitions and Emotional Symptoms

**DOI:** 10.1007/s40653-022-00469-y

**Published:** 2022-08-05

**Authors:** Eleanor M. Bennett, Paul Gray, Jennifer Y. F. Lau

**Affiliations:** 1 Department of Psychology, King’s College London, London, UK; 2 NSW Department of Family and Community Services, Liverpool, NSW Australia

**Keywords:** Maltreatment, Early life adversity, Interpersonal Cognitions, Interpretation biases, Cognitive biases, Interpretation styles, Emotional Disorders

## Abstract

In the general population, negative interpretations of social situations have been associated with risk of developing emotional disorders such as anxiety and depression. Given that childhood maltreatment poses risk for later emotional disorders, this study examined whether interpersonal cognitive style differentiated maltreated adolescents from their non-maltreated peers and correlated with emotional symptoms across each group. Forty-seven maltreated and 28 non-maltreated adolescents were recruited from New South Wales, Australia to complete a battery of questionnaires that assessed interpersonal cognitions and levels of anxiety and depression. Comparable endorsement of threatening interpretations of social situations between maltreated adolescents and their non-maltreated peers across measures was found. Furthermore, an association between anxiety and depressive symptoms and interpretation bias was found within the non-maltreatment group but not the maltreated group. Unlike general population samples, negative cognitions do not associate with emotional symptoms in victims of early maltreatment. More research is needed to investigate the cognitive factors maintaining emotional symptoms in adolescent victims of maltreatment.

## Introduction

 Childhood maltreatment, the physical, sexual, and emotional abuse and neglect of children robustly predicts disruptive, externalising behavioural disorders throughout childhood (Heleniak et al., 2016). Childhood maltreatment also predicts internalising disorders such as depression (Nelson et al., 2017), anxiety (Li et al., 2016), suicidal ideation (Angelakis et al., 2019), and dissociative disorders (Vonderlin et al., 2018), many of which emerge in adolescence and persist into adulthood. Despite this relationship, the mechanisms and processes contributing to emotional disorders remain poorly understood, with many of these disorders deemed ‘treatment resistant’ within this population (Nanni et al., 2012). Recent approaches to delineating mechanisms that mediate between early-life maltreatment and later vulnerability to mental health problems have focused on identifying neurocognitive factors that affect the processing of emotional stimuli (McCrory et al., 2017). Here, we investigated whether negative interpersonal cognitions differentiated adolescent victims of childhood maltreatment from their non-maltreated peers and correlated with anxiety and depression symptoms.

Interpersonal cognitions are defined as a process by which people think about their interactions and relationships with others (Baldwin, 2005). Studies have suggested links between childhood maltreatment and later interpersonal difficulties (Paradis & Boucher, 2010; Reyome, 2010). These abnormalities may arise due to a reduced understanding of social cues, but also due to processing ambiguous social situations differently compared to non-maltreated peers. This is supported by a growing body of research which has noted maltreated youth to both spontaneously infer and numerically rate intentions behind actions of others depicted in vignettes as driven by hostility/threat (Gusler & Jackson, 2017; Kay & Green, 2016; Keil & Price, 2009; Pollak et al., 2000; Price & Glad, 2003; Richey et al., 2016; Shahinfar et al., 2001; Teisl & Cicchetti, 2008). During the experience of adversity, these negative interpersonal cognitions may reap adaptive benefits by alerting the young person of potential danger and allowing them to respond quickly and appropriately to threats (Pollack, 2012). However, when placed in non-threatening environments, atypical cognitive processing may do the opposite, increasing one’s vulnerability to future maladaptive externalising behaviours (Loman & Gunnar, 2010). Consistent with this, interpretations that underscore hostility/threat have been associated with self-rated aggression (Shahinfar et al., 2001), emotion dysregulation (Shields & Cicchetti, 2001), and peer-rated disruptive behaviour (Teisl & Cicchetti, 2008) within maltreated youth.

In non-maltreated adolescents, negative interpersonal cognitions, such as a negative interpretation of ambiguous social situations are frequently used to explain anxiety and depression symptoms (Dearing & Gotlib, 2009; Lau & Pine, 2008; Lau & Waters, 2017; Mogg et al., 2006; Taghavi et al., 2000), emphasising the role cognitive processes play in the development of emotional disorders. Within depression literature, the link between a negative cognitive style and developing both symptoms and diagnoses of depression is well established (Beck, 1987; Clark et al., 1999; Gibb, 2002; Rose & Abramson, 1992). Among subclinical groups, a cognitive bias towards interpreting social situations as rejecting differentiate those most likely to later meet criteria for mood disorders (Kleim et al., 2014; Rude et al., 2002) and are often present in offspring of mothers with anxiety and depression (Dearing & Gotlib, 2009)﻿. It is therefore plausible, given the high rates of internalising symptomology present in maltreated adolescents (Gilbert et al., 2009), that negative interpersonal cognitions and expectations around social rejection situations may also explain anxiety and depressive symptoms within this population. However, no research to date has explored this hypothesis.

Given this gap in research, the present study investigated two research questions: 1. Are there differences in the interpretation of ambiguous social situations in maltreated young people relative to their non-maltreated peers and 2. How do social threat biases associate with anxiety and depression symptoms in maltreated young people? We used three different measures to ascertain interpersonal interpretation style. The Children’s Expectations of Social Behaviours Questionnaire was used to explore expectations regarding ambiguous interactions with others (Rudolph et al., 1995), the Ambiguous Situation Interpretation Scale was administered to look at interpretations of ambiguous social actions of others (Vassilopoulos et al., 2009), and the Perception of Peers and Self questionnaire assessed the perception of others’ actions within social scenarios (Rudolph et al., 1995). We expected maltreated adolescents to experience more negative interpersonal cognitions and thus more negative interpretations of social situations from others’ than those who had not experienced early-life adversity. We also expected these biases to be associated with anxiety and depression symptoms in both groups. Enhancing the knowledge base of vulnerability factors that mediate the relationship between maltreatment and emotional disorders, may translate to and in turn improve treatment target specificity for those experiencing anxiety and depression following early-life victimisation.

## Methods

### Participants

Forty-seven maltreated and 30 comparison adolescents aged 11–17 years old were recruited from 51 households (32 foster care homes, and 18 comparison households) in New South Wales (NSW), Australia. The maltreated participants were recruited from the out‐of‐home care population in NSW where it had been deemed unsafe for them to remain at home. Maltreated participants experienced at least one subtype of abuse and neglect, however, the majority (62%) were exposed to three or more subtypes. The presence/absence and severity of physical abuse (PA), sexual abuse (SA), neglect—failure to provide (FTP), neglect—lack of supervision (LOS), emotional maltreatment (EM), and moral, legal or educational maltreatment (MLE) were coded from file records from the NSW Department of Community Services using the Maltreatment Classification Scheme (MCS) (Barnett et al., 1993). Each recorded notification was coded for each maltreatment subtype and the severity of the abuse/neglect was rated, using 0 to indicate absence of abuse/neglect and 1–5 to indicate severity (Garrido et al., 2011; Litrownik et al., 2005). One quarter of all maltreated participants records were coded independently by two researchers (PG and JL). Inter‐rater reliability for maltreatment subtype presence or absence was calculated using kappas (all = 1.0, except FTP and EM which were unable to be calculated due to the absence of variance between coders). Inter‐rater reliability for maximum severity was also high (ICCs = 0.615). Comparison participants were recruited through local parent networks in the same geographic region. To ensure the comparison group had not experienced adversity, parents completed a brief screening interview. Two comparison participants were excluded after the screener interview identified early-life adversity. The final sample was made up of 47 maltreated adolescents (*M* = 13.42, *SD* = 1.72) and 28 comparison adolescence (*M* = 14.13, *SD* = 1.52). As participants were invited to take part in a study around the effects of early-life experiences on mental health symptoms and their cognitive-affective correlates, power calculations were conducted on the expectation that we would find medium effect sizes across tasks and measures. With the size of group differences expected to fall between 0.50 and 0.70, power was estimated between 0.54 to 0.82 for *p* < 0.05, two-tailed for these participant numbers. Full demographics can be found in Table 1.

**Table 1:** contains the demographics on the maltreated and non-maltreated groups

	Maltreated Sample	Non-maltreated sample
Number (male)	47 (25)	28 (11)
Age	13.42 (1.72)	14.13 (1.52)
IQ	93.12 (10.73)	101.07 (7.88)
Caucasian	62%	79%
Aboriginal Australian	28%	21%
Other/Not specific	10%	-
Physical Abuse	25 (53%)	-
Sexual Abuse	4 (1%)	-
Neglect – failure to provide	44 (94%)	-
Neglect – lack of supervision	40 (85%)	-
Emotional maltreatment	43 (91%)	-

### Measures

#### Sample Characteristics

All participants reported on gender, date of birth, and ethnicity.

#### Interpretation Bias Measures

*The Children’s Expectations of Social Behaviours Questionnaires (CESBQ*; Rudolph et al., 1995) explores negative expectations of imagined social situations using 15 parent and 15 peer vignettes. As we were specifically investigating peer rejection situations, we did not include the parent subscale in our analysis. Participants were asked to imagine themselves in a situation (“You’re on the playground at lunchtime and one of the older kids comes up and starts to pick on you. What do you think the kids in your class might do?”), and to indicate how they believed their peers at school would behave in that situation by choosing a positive, indifferent, or negative response. Responses were scored accordingly: positive = 0, indifferent = 1, and negative = 2. Higher scores reflected more negative interpretations. In the present study, high levels of internal reliability was demonstrated (α = 0.87).

*Ambiguous Situations Interpretations Scale (ASI*; Vassilopoulos et al., 2009) consists of 8 items describing hypothetical social situations with ambiguous outcomes (“You go to your classmate’s house to play together. You ring the bell, but nobody opens the door. Why do you think this happened to you?”). Participants were required to imagine themselves in these situations. Participants were then presented with two possible reasons; one benign (“The classmate is not at home”) and one negative (“He doesn’t want to open the door because I’m boring”) and asked to rate their agreement with each reason on a Likert scale (1 = I would not think that at all, to 5 = I would immediately think that). Responses were summed forming a score for both interpretations (‘negative’ and ‘benign’). For both subscales, high levels of internal reliability was in found in the present study (α = 0.74 Benign; α = 0.77 Negative).

*Perception of Peers and Self (PoPs*; Rudolph et al., 1995) was used to investigate the participant’s perceptions of peers. Participants were presented with 15 statements about ‘others’ (“Other kids cannot be trusted”) and 15 statements about ‘themselves’ (“I am a lot of fun to be with”) and asked to rate the statement on a 4-point Likert scale (where 1 = ‘not at all true’ and 4 = ‘very much true’). As this study was specifically investigating the impact of socially rejecting situations, the ‘self’ subscale was not included in analysis. After appropriate items were reverse scored, scores were summed, with a higher score reflecting a more negative perceptions of ‘others’. The present study noted high levels of internal reliability (α = 0.85).

### Emotional Symptoms

*State-Trait Anxiety Inventory for Child (STAIC*; Spielberger, 1973) is a 20-item self-report measure that asks young people to indicate how much a statement (i.e., I worry too much) describes them (1 = hardly ever, 2 = sometimes, 3 = often). Items are them summed to create an overall anxiety score. Within the present study, Cronbach's α demonstrated high levels of internal reliability (α = 0.82).

*The Children’s Depression Inventory (CDI;* Kovacs, 1985) was used to measure depressive symptoms. Participants were asked to indicate which statement best describes them (i.e., I am sad once in a while, I am often sad, I am sad all the time). An overall depressive symptoms score is then calculated. The CDI displayed high levels of internal consistency within the current study (α = 0.85).

### Procedure

Ethical approval for this study was sought from Research Ethics Committee from institutes in the United Kingdom and Australia. Permission for maltreated adolescents to participate was also granted by the NSW Department of Community Services. Informed consent was sought from all foster carers on behalf of adolescents in their care. Comparison control participants aged over 16 years old were able to give informed consent for themselves, otherwise consent was also sought from their parent/guardian. The measures addressing the current study’s research questions were embedded in a larger battery of questionnaires and tasks assessing various adolescent and parent/guardian features associated with maltreatment and cognitive biases (Gray et al., 2016).

### Statistical Analysis

Interpretation bias measures, PoPs (others subscale) and CESBQ (peers subscale), were compared between maltreatment groups (Maltreated/Non-maltreated) respectively. As Sharpio-Wilk test of normality indicated the data was not normally distributed, non-parametric Mann–Whitney U tests were conducted. As we included both subscales of the interpretation bias measure ASI, and Sharpio-Wilk indicated the data to be normally distributed, a 2 × 2 mixed measures ANOVA was used to examine group differences between maltreatment groups (Maltreated/Non-Maltreated) with the within-subjects factor of Interpretation Type (Negative/Benign) included. The maltreated and comparison participants were matched for age, gender, and household income but not cognitive ability. Therefore, we selected 24 maltreated participants from the larger maltreated sample who were matched to 24 comparison participants on gender, age, cognitive ability, and household income to repeat between‐group comparisons. However, as results for this “matched” sub-sample was similar to the full sample of participants, we present findings for the full 75 participants to maximise statistical power. Contact the corresponding author for more details on the matched sample data. Of note, there was complete data on these questionnaire measures for the full sample.

Next, correlations between the 4 subscales of interpersonal cognitions, anxiety and depression scores were computed for the whole group but also independently for the maltreated and non-maltreatment groups. As Shapiro–Wilk tests of normality indicated the data was not normally distributed, non-parametric Spearmans Rho Correlations were reported. Fishers Z scores were computed to ascertain whether the difference in the magnitude of correlations between the two groups were statistically significant.

## Results

The most prevalent type of adversity within the maltreated sample was ‘neglect – failure to provide’, closely followed by ‘emotional maltreatment’, and ‘neglect – lack of supervision’. Within the maltreated and non-maltreated sample, self-reported depression scores did not meet a clinical cut-off indicative of depression (Kovacs, 1985). However, both samples self-reported mild to moderate levels of anxiety (Spielberger, 1973). Full descriptive statistics for the questionnaire data is presented in Table 2.

**Table 2:** contains the number of completions, range, mean, and standard deviation for all five measures by maltreatment status

Measures		Sample	N	Min	Max	Mean (SD)
ASI	Negative	Non-Maltreated	28	1.25	3.88	2.25 (0.62)
		Maltreated	47	1.00	3.63	2.22 (0.76)
	Benign	Non-Maltreated	28	2.63	4.38	3.53 (0.51)
		Maltreated	47	1.50	5.00	3.40 (0.82)
CESBQ	Peer	Non-Maltreated	28	0.00	13.00	3.82 (4.39)
		Maltreated	47	0.00	24.00	3.40 (5.36)
POPS	Other	Non-Maltreated	28	19.00	46.00	28.38 (6.87)
		Maltreated	47	18.00	55.00	28.97 (7.97)
STAIC	-	Non-Maltreated	28	2	28	13.00 (1.32)
		Maltreated	47	2.11	35	13.65 (0.91)
CDI	-	Non-Maltreated	28	0	27	8.26 (1.52)
		Maltreated	47	0	25	9.06 (0.81)

### Interpersonal Cognitions Between Groups

The 2 × 2 mixed measures ANOVA conducted on ASI data showed no significant main effects of interpretation type (*F*(1,72) = 0.003, *p* = 0.958, η^2^ = 0.013), grouping (*F*(1,72) = 0.277, *p* = 0.600, η^2^ = 0.115) or interaction (*F*(1,72) = 0.018, *p* = 0.893, η^2^ = 0.032). Mann–Whitney U tests were computed on POPs-other and CESBQ-other finding no significant difference in cognitive bias scores between maltreatment groups respectively (*U* = 695, *p* > *0.05*; *U* = 714, *p* > 0.05). Thus no significant difference in interpersonal cognitions between the maltreated and non-maltreated sample emerged.

### Association Between Emotional Symptoms and Interpersonal Cognitions

With the exception of the ASI benign subscale, analysis on the full sample of 75 participants found statistically significant correlations between the three cognitive bias measures for both anxiety (STAIC) and depression (CDI) scores (Table 3). When split by maltreatment status, some of these associations were significantly stronger in the non-maltreated group. The POPS-other subscale was significantly correlated with anxiety (r = 0.611, p < 0.001) and depression scores (r = 0.691, p < 0.001) in non-maltreated participants but not within the maltreated group. However, Fishers Z scores demonstrated that the size of these associations were not statistically different between groups for the POPS-other measure and anxiety (Z = 1.20, p > 0.05) and depression (Z = 1.95, p > 0.05).

**Table 3:** depicts correlations between POPS, CESBQ, ASI and STAIC, CDI for the whole sample, and then split by maltreatment grouping

Cognitive Measures		Whole Sample		Maltreated Group		Non-maltreated group	
		STAIC	CDI	STAIC	CDI	STAIC	CDI
POPS	Other	.424**	.436**	.262	.257	.513**	.633**
CESBQ	Peer	.313**	.533**	.187	.258	.344	.635**
ASI	Negative	.228*	.251*	.230	.204	.446*	.285
	Benign	.040	-.126	.068	.036	-.137	-.454*

^*^Significant at the 0.05 level; **Significant at the 0.01 level

When looking at the association between the ASI cognitive bias measure and anxiety and depression, significant associations between the ASI-negative subscale and anxiety (r = 0.446, *p* < 0.05), and ASI-benign subscale and depression (r = -0.454, *p* < 0.05) was found for the non-maltreated group. No significant associations were found between the maltreated group’s scores. Fishers Z scores showed only the association between the AS-benign and depression score was significantly stronger amongst non-maltreated participants than the maltreatment participants (Z = 2.12, p = 0.034). No other associations were found to significantly differ between the two groups.

Finally, analysis showed the CESBQ-peer subscale to be significantly correlated with depression scores within the non-maltreated group, which was also found to be a non-significantly stronger association than that found in the maltreatment group (Z = 1.96, p = 0.05).

## Discussion

In this study we explored whether interpersonal cognitions differentiated victims of childhood maltreatment from their non-maltreated peers and associated with anxiety and depression symptoms across groups. Surprisingly, no significant differences emerged between the groups across the three cognition measures used. Increased levels of ‘negative’ interpersonal cognitions were associated with elevated anxiety and depression symptoms, however, when split by maltreatment group, this association was either stronger or only remained significant for non-maltreated adolescents.

The tendency to draw rejection-linked interpretations of social situations presented in all three measures were comparable across maltreated and non-maltreated adolescents. Past research does however document that maltreated adolescents spontaneously infer and numerically rate behaviour of others as being derived from hostility (Gusler & Jackson, 2017; Kay & Green, 2016; Pollak et al., 2000; Richey et al., 2016; Shahinfar et al., 2001). Taking the current study’s findings and past research together, it seems that childhood maltreatment elevates the endorsement of hostility (perception of aggression in or provocation by others) rather than the tendency to explain others’ behaviours as being consistent with social rejection. The lack of group differences found in the present study could, however, be explained by the use of vignettes to describe hypothetical social situations. It is possible that the inability of young people to adequately place themselves within the scenarios affected how accurately individual differences in interpretation styles were captured. Perhaps use of pictures or cartoons, or scenarios presented in virtual reality tasks may present more realistic social scenarios for young people to engage with and be more sensitive at detecting group differences in interpretations of rejection. Alternatively, the absence of a group difference in interpretation biases could be explained by characteristics of the maltreated group. The maltreated individuals first entered foster care and thus left adversity prior to their 4^th^ birthday, meaning, that for nearly three-quarters of their life the maltreated sample were likely to be exposed to safer environments, where they could ‘catch up’ in learning, including employing more adaptive, benign interpretation styles (Gregson et al., 2016). As interpretation biases for rejection situations are thought to be modifiable (Lau, 2013), future research tracking interpretation biases from early childhood and across the life span, would provide a more holistic picture of the malleability of cognitive biases within social situations.

Past literature has documented a distinction in hostile interpretation biases elicited by maltreated youth, which may explain the absence of group differences in the present study. Shahinfar et al., (2001) noted adolescents who had experienced physical abuse attributed significantly higher levels of hostility in ambiguous situations when compared to controls, but this was not found for those who had solely experienced emotional abuse or neglect. Around half of the current sample had experience of physical abuse, meaning the remaining sample were classed as maltreated due to experiencing another form of adversity. However, due to sample size restrictions, it was difficult within the sample to find ‘pure’ subtypes of maltreatment, therefore we decided to look at maltreatment as a whole relative to a non-maltreated group. An avenue for future exploration would be whether this distinction of maltreatment subtypes holds for the three interpretation bias measures used in this study.

The association found in the current study between anxiety and depression symptoms and levels of interpretation bias within the non-maltreated sample supports past literature (Dearing & Gotlib, 2009; Lau & Waters, 2017; Mogg et al., 2006), whereby interpreting ambiguous social contexts negatively corresponds to elevated mood symptoms. Consistency in findings is important and holds value for treatments for mood disorders. Despite interpretation styles not differing depending on maltreatment status, associations between mood symptoms and interpretation bias were small and typically non-significant in maltreated adolescents. This disparity suggests the mechanism perpetuating anxiety and depression in previously maltreated adolescents may be different to their non-maltreated peers - namely, it may not be driven by a disposition to explain others’ behavior to be socially rejecting or excluding. It is plausible that facing early-life hardship may result in social situations holding less importance when compared to non-maltreated adolescents, and thus any interpretations of social ambiguities may be less sensitive in predicting mood symptoms. Indeed, Salzinger et al. (1993) found adolescents who had experienced physical abuse had lower peer status, less positive reciprocity with peers, and displayed atypical social networks. Similarly, victims of childhood sexual abuse often display later maladaptive social relationships and disruptions in trust in social situations (Kallstrom-Fuqua et al., 2004; Mullen et al., 1994). Regardless, research has noted that depression within this population is harder to treat than in the general population (Nanni et al., 2012), indicating complex interactions between mood disorder risk factors and maltreatment.

### Limitations

This study had several weaknesses. Despite our best efforts to match groups differences between the maltreated and non-maltreated group remained. Notably, foster care households had lower socio-economic status and were also significantly older than the parents of the non-maltreated adolescents. Although McHugh (2013) documented both factors as being representative of the carer population, this still makes it difficult to interpret between-group designs when comparing such populations with their typically developing peers. Also, the reasons for these young people being placed in care are likely to be complex, for example, involving maladaptive attachment styles with primary caregivers or the presence of parental mental health problems. Although these variables are unmeasured in the present study, they are nonetheless likely to contribute to group differences or the absence of. Another limitation was that the absence of a history of abuse in the comparison participants relied only on parental report and may not accurately reflect maltreatment histories. However, the alternative would have been to use notifications on a child protection register, which would have reflected a significant imposition on the privacy of families and may also not necessarily accurately reflect maltreatment histories since not all instances are reported. Another concern about informants was that both symptoms and cognitive biases were collected through the adolescent’s self-report and subject to reporter bias. Additional parent/guardian reports, and/or additional experimental tasks would overcome this bias and gain a more nuanced picture of symptoms and cognitive biases within this population. Lastly, our sample size was small. While we had > 80% power to detect (medium) between-group effect sizes of 0.70, we were nonetheless, under-powered to detect between-group effect sizes of 0.50–0.60. These findings therefore require replication in larger samples.

Overall, the present study aimed to compare maltreated and non-maltreated youth levels of negative interpersonal cognitions and investigate the association between cognitive styles and symptoms of anxiety and depression in each group. Results found comparable levels of cognitive biases between maltreated adolescents and their non-maltreated peers. Further, an association between mood symptoms and cognitive biases was found within the non-maltreated group but did not exist for the maltreated adolescence. This paper is the first of our knowledge to explore interpretation biases for potential social rejection within a maltreated population. This highlights the need for future research to establish reliable factors that explain the increased vulnerability for mood disorders within this population. This is imperative to further shape treatment and to reduce the highly reported treatment resistance following early-life adversity.

## Data Availability

The data that support the findings of this study are available on request from the corresponding author. The data are not publicly available due to privacy or ethical restrictions.
